# A new genus record and species of *Dromoceryx* Schmidt-Goebel, 1846 (Coleoptera, Carabidae, Lebiini) from Taiwan, with a revised key to species

**DOI:** 10.3897/zookeys.803.29737

**Published:** 2018-12-06

**Authors:** Wesley Hunting, Man-Miao Yang

**Affiliations:** 1 Department of Entomology, National Chung Hsing University, Taichung, 40227 Taiwan National Chung Hsing University Taichung Taiwan

**Keywords:** Carabidae, Dromiina, Lebiini, new species, new record, Taiwan

## Abstract

We describe a new genus record for Taiwan and a new species of the genus *Dromoceryx* Schmidt-Goebel, 1846. We add to the known fauna and distribution of the genus with a description, habitus, genitalic images, as well as a geographic range map for *Dromoceryxnigrofovealis***sp. n.** A revised key to all species of the genus is included.

## Introduction

Until a thorough revision of this genus by Mateu (1984) in French, *Dromoceryx* was considered by many (Atkinson 1890; Bates 1892; Andrewes 1923) to be a likely synonym of *Metabletus* Schmidt-Goebel, 1846. Mateu treated the three known species and cited the structure of the mentum (with a median tooth), glossal sclerite, unique elytral macula pattern, and genitalic form of both male and female as characteristics that define *Dromoceryx* as a genus. Other than an apparent South (India) and South-east (Vietnam) Asian distribution, little else is known about the natural history of this group. Here, we describe a new species of the genus *Dromoceryx* based on the examination of several specimens recently collected in Taiwan.

## Materials and methods

### Material

This work is based on the study of 36 specimens. Adult specimens were collected by or borrowed from various institutions listed below, along with a four-letter coden used to identify sources of specimens (Arnett et al. 1993). The names of the curators of these collections appear in parentheses below.

**CMNH**Section of Invertebrate Zoology, Carnegie Museum of Natural History, 4400 Forbes Avenue, Pittsburgh, Pennsylvania, U.S.A. 15213-4080 (R. L. Davidson).

**NCHU**Department of Entomology, National Chung Hsing University, Taichung City 402, Taiwan (Man-Miao Yang).

**NMNS**National Museum of Natural Science, One Guancian Road Taichung City 404, Taiwan (Jing-Fu Tsai).

**TARI**Insect Collection of the Taiwan Agricultural Research Institute, Wufeng District, Taichung City 41362, Taiwan (Chi-Feng Lee).

**UASM**E.H. Strickland Entomology Museum, University of Alberta, Edmonton, Alberta, Canada, T6G 2H1 (Danny Shpeley).

### Methods

***Fieldwork.*** We first encountered this species when Yen-Chiu Lan (University of Kang Ning, Tainan) provided specimens collected from her 2010 faunistic study on the insects of Kenting National Park, Pingtung County, Taiwan. Our examination of this material indicated that it was both a new genus record for Taiwan and a new species for the genus *Dromoceryx*. After scouring other museums for specimens of this species, it was clear that little was known about the habitat preferences or biology of *Dromoceryx* species other than that they readily came to ultraviolet light and light traps. Because of this, we were eager to locate more individuals in the field. Adults of this species had also been previously collected from the Liouguei Research Center of southern Taiwan. In April of 2014, Yi-Ming Weng, Dash Hwang, and Wesley Hunting, went there for several days to try and locate this seemingly uncommon insect species. During that time we were able to collect three individuals, two specimens from a mercury vapour light sheet and one in mixed primary forest on deadwood.

***Preparation and examination of adults.*** Standard methods were used for mounting, dissecting, and preparing genitalia, among other technical procedures (Ball and Hilchie 1983; Frania and Ball 2007). Genitalia and other small structures were preserved in glycerine and stored in microvials that were pinned beneath the specimen from which they had been removed.

**Figure 1.**
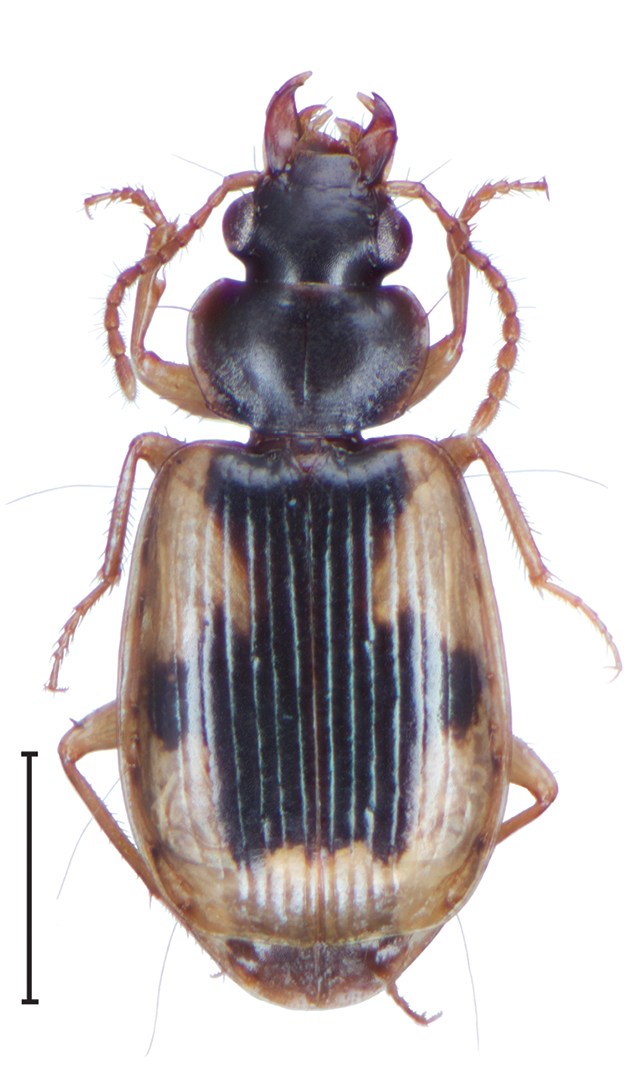
Dorsal habitus and color pattern of *Dromoceryxnigrofovealis*, new species. Scale bar: 1 mm.

**Figure 2.**
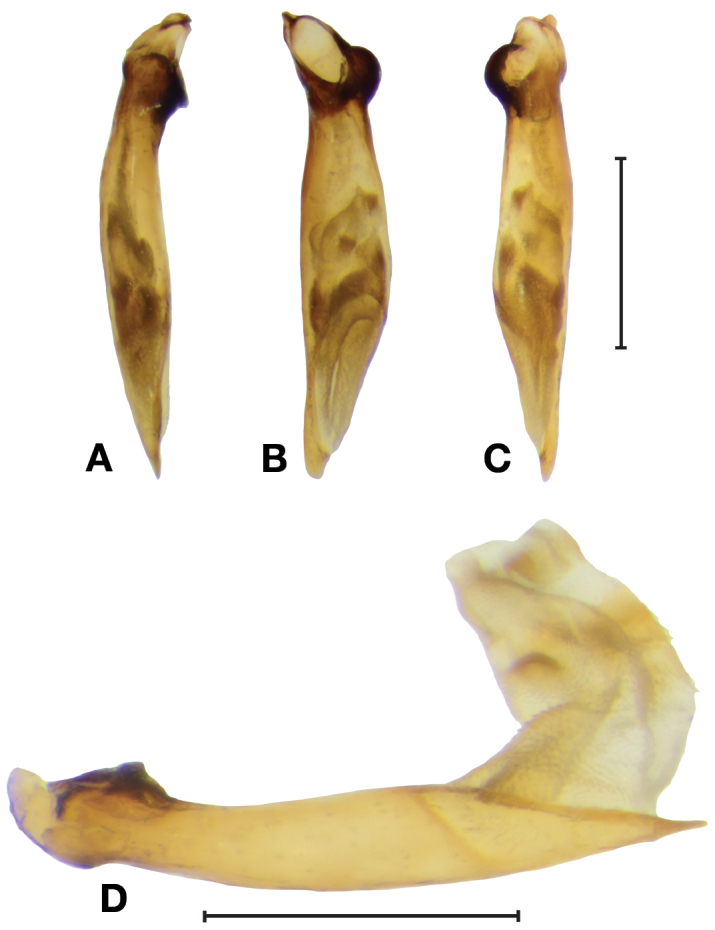
Digital images of male genitalia of *Dromoceryxnigrofovealis*, new species **A** Right lateral aspect **B** Ventral aspect **C** Left lateral aspect **D** Right lateral aspect with endophallus everted. Scale bars: 0.5 mm.

**Figure 3.**
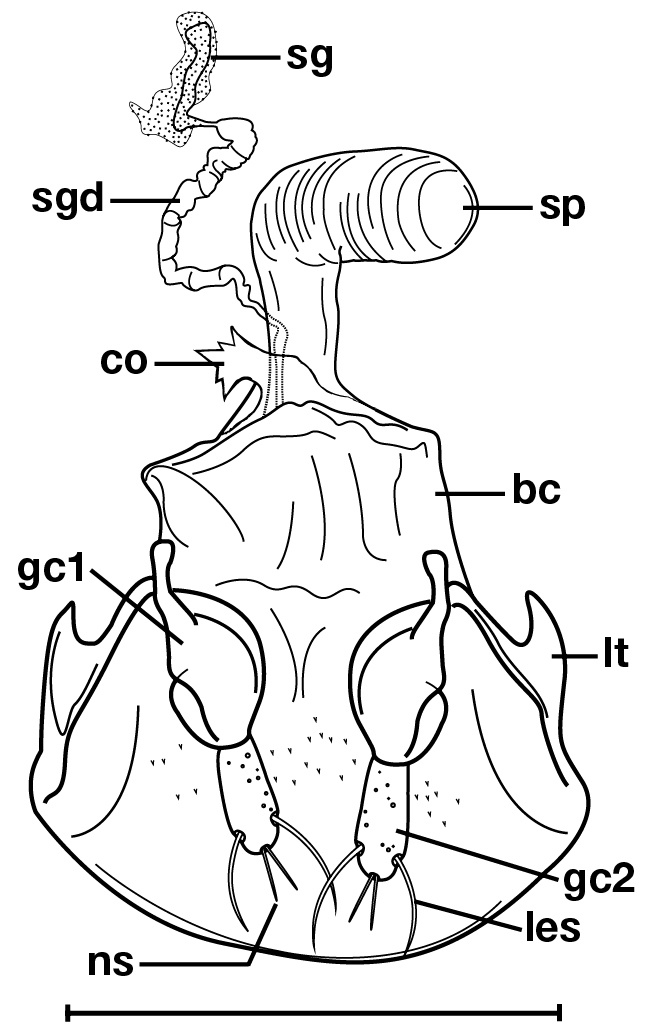
Line drawing of the female reproductive tract of *Dromoceryxnigrofovealis*, new species. Legend: **bc** bursa copulatrix; **co** common oviduct; **gc1** gonocoxite 1; **gc2** gonocoxite 2; **les** lateral ensiform setae; **lt** lateral tergite; **ns** nematiform setae; **sg** spermathecal gland; **sgd** spermathecal gland duct; **sp** spermatheca. Scale bar: 0.5 mm.

***Images and illustrations.*** A photograph of species habitus (Fig. 1) was taken using a Nikon D7100 fitted with an AF-S VR Micro-NIKKOR 105mm f/2.8G IF-ED lens and mounted on a copy stand. Photographs of genitalia (Fig. 2a–d) were taken with a Nikon D7100 mounted on a Olympus SZX16 trinocular stereoscopic microscope and layered together using Zerene Stacker (Zerene Systems LLC, Richland, WA). A line drawing of the female genital tract (Fig. 3) was prepared by taking photographs with a Nikon D7100 and then importing them into Adobe Illustrator 11.0 (Adobe Systems, Inc., Mountainview, CA). Plates were also prepared using Adobe Illustrator 11.0.

The Geographic range map was prepared using a modified map from Ginkgo Maps (http://www.ginkgomaps.com); projection used is NAD Lambert Conformal Conic, 1983.

***Measurements.*** Measurements were made at 25× with a Wild M5 stereoscopic microscope fitted with an ocular micrometer. Various measurements are expressed in the text by abbreviations, as used by Ball and Shpeley (2005) and Hunting (2013):

**HL** Length of head, measured on left side, from base of left mandible to posterior margin of compound eye.

**HW** Width of head, maximum transverse distance across head, including eyes.

**PL** Length of pronotum along midline.

**PWM** Maximum width of pronotum.

**ML** Metepisternum length.

**MW** Metepisternum width.

**EL** Length of elytra from basal ridge to apex.

**EW** Maximum width of elytra.

**OBL** Overall body length.

The shape of the head and pronotum is shown by the ratio of the width over length (**HW/HL**; **PWM/PL**, **ML/MW**), and elytral shape is indicated by the ratio of the length to the width (**EL/EW**).

To indicate the range of body size of each species, the overall body length (**OBL**) was measured from the apex of the extended mandibles to the apex of the elytra of both the largest and smallest individuals of each species (Frania and Ball 2007).

The size of male genitalia was determined by drawing a straight line between the apical area and the basal lobe of the phallus. The size of female genitalia was determined by drawing a straight line across the outside margin of widest portion of left lateral tergite to outside margin of widest portion of right lateral tergite.

## Systematic zoology

### Order Coleoptera Linnaeus, 1758

#### Family Carabidae Latreille, 1802

##### Subfamily Lebiinae Bonelli, 1810

###### Genus Dromoceryx

Taxon classificationAnimaliaColeopteraCarabidae

Schmidt-Goebel, 1846

####### Type species.

*Dromoceryxdorsalis* Schmidt-Goebel, 1846: 40–41. Atkinson 1890: 75; Andrewes 1923: 18; Mateu 1984: 404; Lorenz 2005: 478.

####### Taxonomic note.

The type of *D.dorsalis* is in the National Museum of Natural History (NMPC), Prague, Czech Republic. According to Andrewes (1923) and Mateu (1984), it is a single specimen with no antennae or palpi and is in otherwise poor condition.

####### Recognition.

This genus is distinguished from others by the following combination of characters: Broad and somewhat flattened body. Small size: 3.5–4.5 mm. Glossal sclerite broad, with narrow latero-apical lobes, four setae visible at apex, two longer seta more laterally and two shorter setae more medially. Mentum with single shallow tooth. Head and pronotum brunneous to piceous, elytral disc testaceous with black macula. Gonocoxite 2 slightly spatulate, broadly rounded at apex; two lateral ensiform setae, one on each side, seta-like as opposed to spine-like, two nematiform setae. For a detailed account and figures of the male and female genitalia of the already described species of *Dromoceryx*, see Mateu (1984).

##### Key to species of the genus *Dromoceryx*

**Table:** 

1	Relatively large (4.4–4.5 mm). Elytral disc rufous to red, macula in apical portion of elytra, not contacted to apical third of elytral suture. India, Chennai (Madras)	***D.magnus* Mateu**
–	Smaller, < 4.1 mm. Elytral disc testaceous, macula covering, at least, basal two-thirds of elytral suture. India and elsewhere	**2**
2	Overall body length 3.0–3.2 mm. Elytra disc with isodiametric sculpticells. India, Chennai (Madras)	***D.flavocircumdatus* Mateu**
–	Overall body length > 3.4 mm. Elytra disc with transverse sculpticells	**3**
3	Elytra with foveae of umbilical setae piceous (Fig. 1). Disc of pronotum brunneo-piceous to piceous. Taiwan	***D.nigrofovealis* sp. n.**
–	Elytra with foveae of umbilical setae testaceous. Disc of pronotum brunneous. India,Vietnam	***D.dorsalis* Schmidt-Goebel**

##### Dromoceryx nigrofovealis

Taxon classificationAnimaliaColeopteraCarabidae

sp. n.

http://zoobank.org/4FBD41B7-BA20-484E-9BB6-757A6F05B4FD

**Figure 4.**
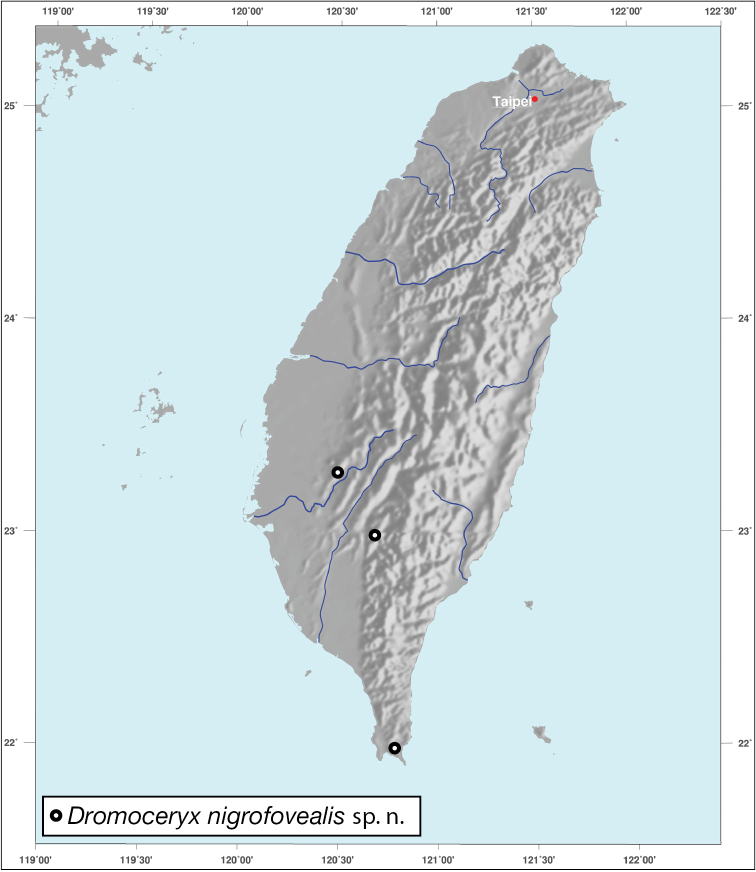
Map showing known localities for *Dromoceryxnigrofovealis*, new species, in Taiwan.

Figs 1 , 2A–D , 3 , 4

###### Etymology.

The name of this species refers to the foveae of the lateral umbilical seta, which is piceous to black.

###### Type material.

HOLOTYPE, male, labeled: “TAIWAN: Kaohshiung City / Maolin dist., Chung-Shin vill. / Liouguei Research Center / 22.9709N, 120.6822E”; “m.v. light sheet / 670m, Acc. Ti-209a / April 14, 2014, Y. Weng / D. Hwang & W. Hunting” [NCHU]. 35 PARATYPES, sex and label data follows. 1 male, labeled same as holotype [NCHU]. 1 female, “TAIWAN: Kaohshiung City / Maolin dist., Chung-Shin vill. / Liouguei Research Center / 22.9709N, 120.6822E”; “hand coll., April 15, 2014 / 640m, Acc. Ti-210d / Y.M. Weng, D. Hwang / & W. M. Hunting” [NCHU]. 2 males, 4 females, “TAIWAN: Kaohsing. / Shanping., 640 m / 1–10 April 1998/ R. Davidson, J. Rawlins / C. Young” [CMNH]. 3 males, 2 females, “TAIWAN: Kaohsing./ Shanping., 640 m / 21–30 April 1998 / C. Young, R. Davidson / J. Rawlins” [CMNH]. 4 males, 3 females, “TAIWAN Hengchun / Kenting Park/ IV / 7–8/2004 / C.S. Lin & W.T. Yang / UV Light trap” [NMNS]. 1 male, 1 female, “TAIWAN Hengchun/ Kenting Park / II/16–17/2005 / C.S. Lin & W.T. Yang / UV Light trap” [NMNS]. 4 males, 2 females, “TAIWAN: Tainan Co. / Mt. Kantou Trail / (崁頭山步道) by light/ 2014.III.29. leg. 賴保成 / 23°15'36.55"N, 120°30'00.85"E [NCHU]. 4 males, 3 females, “ TAIWAN: Pingdong Co. / Kenting Forest Research / Area (墾丁森林遊樂區) / 2010.V.15 by light”; “2010-5-15-Kenting Forest / Recreation Area (墾丁森林/ 遊樂區) – Light trap / -Coleoptera- 53”; leg. 邱垂生 & 藍艷秋/ 21°57'48.6"N, 120°48'47.9"E/ #372” [LAN].

###### Type Locality.

Maolin District, Kaohshiung City, Taiwan.

###### Diagnosis.

This species is readily distinguished from other *Dromoceryx* species by the combination of: head and pronotum piceous to black and lateral margins with foveae of umbilical setae piceous. Individuals of this species also have an elytral macula pattern that is less variable when compared to specimens of *D.dorsalis* (Mateu 1984).

###### Description.

OBL 3.52–4.08 mm. Length (*n* = 10 males, 10 females): head 0.32–0.44, pronotum 0.60–0.72, elytra 2.10–2.42, metepisternum 0.56–0.60 mm; width of head 0.82–0.96, of pronotum 1.04–1.20, of elytra 1.66–1.92, of metepisternum 0.32–0.40 mm.

***Body proportions.***HW/HL 2.10–2.56; PWM/PL 1.63–1.77; EL/EW 1.25–1.33; ML/MW 1.50–1.75 mm.

***Color.*** Fig. 1. Dorsum of head piceous, clypeus with base piceous, apical margin brunneous, labrum brunneo-piceous to brunneous, darker centrally; palpi and antennae brunneo-testaceous to brunneous; elytral disc piceous to black, margins brunneous, paler; elytral disc testaceous, lateral margins with foveae of several umbilical setae piceous, disc with two large piceous macula, joined along suture to appear as one large macula, macula long, extending from base to apical 1/6^th^ of elytra. At base of elytra, from suture to stria 6, narrowing to stria 4 in basal 1/3^rd^ and then expanding to interval 8 medially, constricting again just beyond 2/3^rds^ length to stria 5; ventral surface distinctive, apex of prosternal and mesosternal coxal process testaceous, metepisternum and pregentinal sterna III and IV testaceous medially, apical margin of pregenital sternum VII brunneous to testaceous, proepipleuron testaceous, legs testaceous, all other surfaces brunneo-piceous to piceous.

***Microsculpture.*** Dorsum of head with mesh pattern isodiametric to slightly stretched longitudinally; pronotum with microsculpture almost isodiametric medially to somewhat transverse laterally, cells 1.5–2× wider that long; elytra with sculpticells transverse; ventral surfaces with microsculpture transverse.

***Macrosculpture and pilosity.*** Dorsum of head smooth, with a few very fine punctures, hardly visible at 50×. Pronotum smooth, with very fine, randomly scattered setigerous punctures, hardly visible at 50×; elytral intervals with ± single row of very fine, setigerous punctures along length, hardly visible at 50×; stria with few faint punctations, setae not visible at 50×; ventral surface with very fine, randomly scattered setigerous punctures.

***Fixed setae.*** Two pairs of supraorbital setae; clypeus with two lateral setae; labrum with six setae along apical margin; pronotum with two setae along each margin, one at base of lateral margin and one on lateral margin at pronotum maximal width; elytra with two setae in interval 3, one seta just before mid-length, one seta in apical 1/3^rd^; 11–12 lateral (umbilical) setae in interval 9; two setae on each of abdominal sterna III to VI; two setae along apical margin of sternum VII in males, females with four setae near apical margin of sternum VII, medially setae much shorter and finer than outer setae.

***Luster.*** Dorsum of head and pronotum moderately dull; elytra moderately glossy; ventral surface moderately glossy.

***Head.*** Mandibles short, with wide base; labrum wider than long, rectangular; mentum with shallow tooth; eyes somewhat convex.

***Pronotum.*** Anterior transverse impression very shallow; posterior transverse impression very shallow; median longitudinal impression moderately shallow; disc moderately flat, basal angles obtuse, lateral margins broadly rounded, margins narrow.

***Elytra.*** Intervals moderately flat, striae moderately impressed; elytral apices truncate.

***Hind wings.*** Macropterous.

***Legs.*** Claws pectinate, 4 or 5 denticles per claw. Male protarsi with adhesive vestiture ventrally, two rows of squamo-setae on tarsomeres 1–3 of fore-leg.

***Male genitalia.*** Fig. 2A–D. Length 0.84–0.92 mm. Ostium catopic, long, slightly more to left side in ventral view; phallus cylindrical, left side narrowing from mid-length to apex, right side relatively straight in ventral view, apex narrow, rounded, sharply pointed in lateral view; endophallus short and wide, several patches with microtrichia slightly enlarged and more sclerotized than remaining surface, visible in non-everted specimens.

***Female genitalia.*** Fig. 3. Width 0.56–0.64 mm. Gonocoxite 2 (**gc2**) slightly spatulate, broadly rounded at apex; two lateral ensiform setae (**les**), one on each side, seta-like as opposed to spine-like, two nematiform setae (**ns**) at apex; one spermatheca (**sp1**), cylindrical and elongate, right angled at mid-length, ribbed texture from mid-length to apex; one spermathecal accessory gland (**sg**), spermathecal gland duct (**sgd**) with irregular width along length, attachment site at base of spermatheca.

###### Habitat, habits and seasonal occurrence.

The known elevational range of *D.nigrofovealis* is from 240 to 670 m. Adults of this species live in mixed primary and secondary forests. Adults are crepuscular or nocturnal and readily come to light. All known specimens have been collected from February to May. Collecting methods include ultraviolet light, mercury vapour light sheet, incandescent light, and hand collecting.

###### Geographical distribution.

To date, this species is known from a few localities in the southern third portion of Taiwan (Fig. 4).

## Supplementary Material

XML Treatment for Dromoceryx

XML Treatment for Dromoceryx nigrofovealis
